# Supplementary material for: Myofibroblast Ccn3 is regulated by Yap and Wwtr1 and contributes to adverse cardiac outcomes
Source: Front Cardiovasc Med. 2023 Mar 14;10:1142612. doi: 10.3389/fcvm.2023.1142612 (PMC10043314; doi:10.3389/fcvm.2023.1142612)
Supplement: Supplementary file 6 [file Datasheet2.pdf]

```
#### Obtain references ####

# START BASH CODES #

#NCBI GRCm39 and Annotation Release 109 (downloaded Nov 2021)
wget
https://ftp.ncbi.nlm.nih.gov/genomes/all/GCF/000/001/635/GCF_000001635.27_GRCm39/
GCF_000001635.27_GRCm39_genomic.fna.gz
wget
https://ftp.ncbi.nlm.nih.gov/genomes/all/GCF/000/001/635/GCF_000001635.27_GRCm39/
GCF_000001635.27_GRCm39_genomic.gff.gz
wget
https://ftp.ncbi.nlm.nih.gov/genomes/all/GCF/000/001/635/GCF_000001635.27_GRCm39/
GCF_000001635.27_GRCm39_assembly_report.txt

# The EGFP sequence (GenBank: U55761.1) was manually obtained from
# https://www.ncbi.nlm.nih.gov/nuccore/1377908 by getting the  the complete record
# as a FASTA file (Send to: Complete Record, Destination: File, Format: FASTA;
named it "egfp-full-sequence.fasta"),
# the coding region as a FASTA file (Send to: Coding Sequences, Format: FASTA
Nucleotide; named it "egfp-coding-sequence.fasta"),
# and downloaded the full sequence as a GFF3 file (Send to: Complete Record,
Destination: File, Format: GFF3; named it "egfp-full-sequence.gff3").

# The SA-betageo was obtained from https://www.addgene.org/browse/sequence/9965/ by

wget https://media.addgene.org/snapgene-media/v1.7.9-0-g88a3305/sequences/9965/
b98a6f6e-931d-4efb-9526-2ab541977700/addgene-plasmid-21709-sequence-9965.gbk

# It was manually converted to fasta format using
https://www.bioinformatics.org/sms2/genbank_fasta.html
# and then copied/pasted the output into bGEO-full-sequence.fasta.
# The information in the https://www.addgene.org/browse/sequence/9965/ features tab
was used
# to manually make a .gtf file named "bGEO-full-sequence_for_CellRanger.gtf" for
genes lacZ,
# NeoR/KanR and AmpR.

#### Modify gff files ####

# NCBI's gff needs lots of modifications to be able to be used in cellranger mkref:
#
# 1. Put EntrezID in gene_id attribute
# 2. Put symbols in gene_name attribute
# 3. Remove entries without transcript_id values
# 4. Remove entries with strand *
# 5. Remove Y versions of PAR genes
# 6. Export as gtf format

# END BASH CODES #

# START R CODES #

# These were done in R v4.0.3 using these codes:

args <- c("GCF_000001635.27_GRCm39_genomic_109.gff",
          "GCF_000001635.27_GRCm39_genomic_109_for_CellRanger.gtf")
```

```
library(rtracklayer)

gff0 <- rtracklayer::import(args[1])

#  1. Put EntrezID in gene_id attribute

temp <- sapply(gff0$Dbxref, function(x) x[grep("GeneID", x)])
#If didn't have GeneID, switch to NA
temp2 <- sapply(temp, length)
if(any(temp2 == 0))
  temp[temp2 == 0] <- NA
#If have more than one GeneID, keep first one
if(any(temp2 > 1))
  temp[temp2 > 1] <- lapply(temp[temp2 > 1], function(x) x[1])
#Put GeneID in attribute named gene_id
gff0$gene_id <- unlist(temp)
#If no GeneID, put in value from product attribute
if(sum(is.na(gff0$gene_id)) > 0)
  gff0$gene_id[is.na(gff0$gene_id)] <- gff0$product[is.na(gff0$gene_id)]

# If transcript_id is NA, put in gene_id (to keep mitochondrial genes)

gff0$transcript_id[is.na(gff0$transcript_id)] <-
gff0$gene_id[is.na(gff0$transcript_id)]


# 2. Put symbols in gene_name attribute

gff0$gene_name <- gff0$gene


# 3. Remove entries without transcript_id values

gff.exon <- gff0[gff0$type == "exon"]
temp <- gff.exon[is.na(gff.exon$transcript_id)]
gff1 <- gff0[!gff0$gene_id %in% temp$gene_id]


# 4. Remove entries with strand *

temp <- gff1[strand(gff1) %in% "*"]
gff1 <- gff1[!gff1$gene_id %in% temp$gene_id]


# 5. Remove Y versions of PAR genes

temp <- gff1[gff1$type %in% c("gene", "exon")]
tempX <- temp[seqnames(temp) %in% "NC_000086.8"]
tempY <- temp[seqnames(temp) %in% "NC_000087.8"]


tempX <- tempX[!duplicated(tempX$gene_id)]
tempY <- tempY[!duplicated(tempY$gene_id)]

temp <- tempY[tempY$gene_id %in% intersect(tempX$gene_id, tempY$gene_id)]
$transcript_id
gff1 <- gff1[!gff1$transcript_id %in% temp]
```

```
# 6. Finally, export gff in gtf format

rtracklayer::export(gff1, args[2], format = "gtf")


gff2 <- gff1[gff1$type == "exon"]
gff2 <- gff2[!duplicated(gff2$gene_id) & !is.na(gff2$gene_id)]

gtf_gene_df <- as.data.frame(gff2)

chrnames <- read.delim("GCF_000001635.27_GRCm39_assembly_report.txt",
                       comment.char = "#", header = FALSE)

names(chrnames) <- c("SequenceName",
                     "SequenceRole",
                     "Assigned_Molecule",
                     "Assigned_Type",
                     "GenBank_Accn",
                     "Relationship",
                     "RefSeq_Accn",
                     "Assembly_Unit",
                     "Sequence_Length",
                     "UCSC_name")

levels(gtf_gene_df$seqnames) <-
chrnames$SequenceName[match(levels(gtf_gene_df$seqnames), chrnames$RefSeq_Accn)]

temp <- gtf_gene_df[,c("gene_id","gene_name","product","seqnames","gbkey")]
names(temp)[c(4, 5)] <- c("chr","gene_type")

write.table(temp, file = "Gene_Info_GRCm39_annot109.txt", row.names = FALSE, quote
= FALSE, sep = "\t")

#Also write out just MT genes

write.table(temp[temp$chr == "MT","gene_name"], file =
"MT_symbols_GRCm39_annot109.txt",row.names=FALSE,
            col.names = FALSE, quote = FALSE, sep = "\t")


#Also modify eGFP gff file:

gff0 <- rtracklayer::import("egfp-full-sequence.gff3")

# 1. Change CDS to exon

levels(gff0$type)[3] <- "exon"

#2. Put gene-level ID in gene_id attribute and gene_name and transcript_id

gff0$gene_id <- c("Egfp","Egfp","Egfp", "neomycin")
gff0$gene_name <- c("Egfp","Egfp","Egfp", "neomycin")
gff0$transcript_id <- c("Egfp","Egfp","Egfp", "neomycin")

# 3. Export as gtf format

export(gff0, "egfp-full-sequence_for_CellRanger.gtf", format = "gtf")
```

```
# END R CODES #

# START BASH CODES #

# Fix egfp  and bGEO FASTA line lengths

module load seqtk/1.3-IGB-gcc-8.2.0
seqtk seq -l 80 egfp-coding-sequence.fasta > egfp-coding-sequence-80.fasta
seqtk seq -l 80 egfp-full-sequence.fasta > egfp-full-sequence-80.fasta
seqtk seq -l 80 bGEO-full-sequence.fasta > bGEO-full-sequence-80.fasta



#### Merge reference files ####

cat GCF_000001635.27_GRCm39_genomic.fna egfp-full-sequence-80.fasta bGEO-full-
sequence-80.fasta > GCF_000001635.27_GRCm39_genomic_egfp_bGEO.fna

cat GCF_000001635.27_GRCm39_genomic_109_for_CellRanger.gtf >
GCF_000001635.27_GRCm39_genomic_109_and_EGFP_bGEO_for_CellRanger.gtf
grep -vP "^##" egfp-full-sequence_for_CellRanger.gtf >>
GCF_000001635.27_GRCm39_genomic_109_and_EGFP_bGEO_for_CellRanger.gtf
grep -vP "^##" bGEO-full-sequence_for_CellRanger.gtf >>
GCF_000001635.27_GRCm39_genomic_109_and_EGFP_bGEO_for_CellRanger.gtf


#### Make Custom Reference ####

# Make custom reference using following script:

#!/bin/bash
#SBATCH -n 24
#SBATCH --mem=50G

module load cellranger/6.1.1

cellranger mkref \
--genome=cellranger_6.1.1_ncbi_GRCm39_109_and_EGFP_bGEO \
--fasta=GCF_000001635.27_GRCm39_genomic_egfp_bGEO.fna \
--genes=GCF_000001635.27_GRCm39_genomic_109_and_EGFP_bGEO_for_CellRanger.gtf \
--ref-version='ncbi_GRCm39_anno109' \
--nthreads=$SLURM_NTASKS \
--memgb=50


# END BASH CODES#

# START R CODES #

#### Seurat Analyses ####

# These were done in R v4.1.2

library(dittoSeq)
library(tidyverse)
library(Seurat)
library(sctransform)
library(scater)
library(scran)
```

```
library(patchwork)


#### read in data and sample info ####

aggr.data <- Read10X(data.dir = "Aggr_filtered_feature_bc_matrix/")
sampOrder <- read.csv("aggregation.csv")
allcells_prefilt <- CreateSeuratObject(counts = aggr.data, min.cells = 20,
                               project = "omeara", names.field = 2, names.delim =
"-")
levels(allcells_prefilt$orig.ident) <- sampOrder$sample_id
Idents(allcells_prefilt) <- "orig.ident"
rm(aggr.data)
gc()

# #### Find %UMIs in MT genes ####

MT.genes <- read.delim("MT_symbols_GRCm39_annot109.txt", header = FALSE)[,1]
MT.genes <- MT.genes[MT.genes %in% rownames(allcells_prefilt)]
allcells_prefilt[["percent.mt"]] <- PercentageFeatureSet(allcells_prefilt, features
= MT.genes)


# #### Norm, cluster and UMAP - prefiltering ####

allcells_prefilt <- SCTransform(allcells_prefilt,
                        method = "glmGamPoi",
                        vars.to.regress = "percent.mt",
                        return.only.var.genes = FALSE)
allcells_prefilt <- RunPCA(allcells_prefilt, verbose = FALSE)
allcells_prefilt <- FindNeighbors(allcells_prefilt, dims = 1:40, verbose = FALSE)
allcells_prefilt <- FindClusters(allcells_prefilt, verbose = TRUE, resolution =
0.5)
allcells_prefilt <- RunUMAP(allcells_prefilt,
                    dims = 1:40,
                    verbose = FALSE)

#### Find UMI counts of focal genes ####

temp <- GetAssayData(object = allcells_prefilt, slot = "counts", assay = "RNA")

allcells_prefilt$focalgenes.nCount <- colSums(temp[c("Yap1","Wwtr1","Postn"),])
allcells_prefilt$anyFocal <- ifelse(allcells_prefilt$focalgenes.nCount > 1, "yes",
"no")

allcells_prefilt$any_Yap <- ifelse (temp["Yap1",] > 0, "Yap.yes", "Yap.no")
allcells_prefilt$any_Wwtr <- ifelse (temp["Wwtr1",] > 0, "Wwtr.yes", "Wwtr.no")
allcells_prefilt$any_Postn <- ifelse (temp["Postn",] > 0, "Postn.yes", "Postn.no")
allcells_prefilt$jointFocal <- paste(allcells_prefilt$any_Yap,
allcells_prefilt$any_Wwtr, allcells_prefilt$any_Postn, sep ="_")

allcells_prefilt$Yap_group <- allcells_prefilt$any_Yap
allcells_prefilt$Yap_group[allcells_prefilt$jointFocal %in%
c("Yap.no_Wwtr.no_Postn.yes",

"Yap.no_Wwtr.yes_Postn.no",

"Yap.no_Wwtr.yes_Postn.yes")] <- "other.yes"
```

```
#### Filter out cells ####

allcells_prefilt$prefilt_clusters <- allcells_prefilt$seurat_clusters

allcells <- allcells_prefilt[,!allcells_prefilt$seurat_clusters %in% c(0,8,9)]
Idents(allcells) <- "orig.ident"


#### Find QC thresholds based on 6 MAD ####

temp1 <- scater::isOutlier(allcells$percent.mt, nmads = 6, type = "higher")
mt.threshold <- min(allcells$percent.mt[temp1])
temp1 <- scater::isOutlier(allcells$nCount_RNA, nmads = 6, type = "higher")
UMI.threshold <- min(allcells$nCount_RNA[temp1])


#only keep cells that are under both mt.threshold AND UMI.threshold

n.filtered <- ncol(allcells) - sum(allcells$percent.mt < mt.threshold &
                                     allcells$nCount_RNA < UMI.threshold)


allcells <- subset(allcells, subset = percent.mt < mt.threshold &
                     nCount_RNA < UMI.threshold )


#### Re-run pipeline ####

allcells <- SCTransform(allcells,
                        method = "glmGamPoi",
                        vars.to.regress = "percent.mt",
                        return.only.var.genes = FALSE)
allcells <- RunPCA(allcells, verbose = FALSE)
allcells <- FindNeighbors(allcells, dims = 1:40, verbose = FALSE)
allcells <- FindClusters(allcells, verbose = TRUE, resolution = 0.5)
allcells <- RunUMAP(allcells,
                    dims = 1:40,
                    verbose = FALSE)


#### Find cluster markers ####

Idents(allcells) <- "seurat_clusters"
DefaultAssay(allcells) <- "SCT"
all_markers <- FindAllMarkers(allcells, verbose = TRUE,
                              assay = "SCT",
                              slot = "data",
                              logfc.threshold = 0.25)

top10 <- all_markers %>% group_by(cluster) %>%
  slice_min(order_by =  p_val, n = 10, with_ties = FALSE) %>%
  ungroup()


#### Compare Yap and RGFP per cluster ####

allcells$short <- as.character(allcells$orig.ident) %>% strsplit( "_") %>%
sapply(function(x) x[1])
allcells$trt_cluster <- paste(allcells$short, allcells$seurat_clusters, sep = "_")
```

```
allcells$trt_cluster <- factor(allcells$trt_cluster, levels =
paste(c("RGFP","Yap"), rep(0:18, each = 2), sep = "_"))

Yap_vs_RGFP <- NULL

Idents(allcells) <- "trt_cluster"
#Test all clusters except 16 (17th in 0:18) - only 3 cells in Yap
for(i in (0:18)[-17]) {
  temp <- FindMarkers(allcells,
                      ident.1 = paste0("Yap_", i),
                      ident.2 = paste0("RGFP_", i),
                      verbose = FALSE,
                      assay = "SCT",
                      slot = "data",
                      min.pct = 0.25,
                      logfc.threshold = 0.25)
    temp <- rownames_to_column(temp, var = "gene")
    temp$cluster <- i
    Yap_vs_RGFP <- rbind(Yap_vs_RGFP, temp)
write.csv(temp,
          file = paste0("Yap_vs_RGFP_cluster", i, ".csv"),
          row.names = FALSE)

}

top10_YvR <- Yap_vs_RGFP %>% group_by(cluster) %>%
  slice_min(order_by =  p_val, n = 10, with_ties = FALSE) %>%
  ungroup()



#### subset to cluster 5 ####

cluster5 <- allcells[,allcells$seurat_clusters == 5]

#Run pipeline to find sub-clusters

cluster5 <- SCTransform(cluster5,
                        method = "glmGamPoi",
                        vars.to.regress = "percent.mt",
                        return.only.var.genes = FALSE)
cluster5 <- RunPCA(cluster5, verbose = FALSE)
cluster5 <- FindNeighbors(cluster5, dims = 1:40, verbose = FALSE)
cluster5 <- FindClusters(cluster5, verbose = TRUE, resolution = 0.5)
cluster5 <- RunUMAP(cluster5,
                    dims = 1:40,
                    verbose = FALSE)



#Find new markers

Idents(cluster5) <- "seurat_clusters"
DefaultAssay(cluster5) <- "SCT"
c5_markers <- FindAllMarkers(cluster5, verbose = TRUE,
                              assay = "SCT",
                              slot = "data",
                              logfc.threshold = 0.25)
```

```
#Find trt differences in clusters 0, 3, 4 and 5 only

cluster5$trt_cluster <- paste(cluster5$short, cluster5$seurat_clusters, sep = "_")

Idents(cluster5) <- "trt_cluster"

c5.c0.YvR <- FindMarkers(cluster5,
                         ident.1 = "Yap_0",
                         ident.2 = "RGFP_0",
                         verbose = FALSE,
                         assay = "SCT",
                         slot = "data",
                         min.pct = 0,
                         logfc.threshold = 0)
c5.c0.YvR <- rownames_to_column(c5.c0.YvR, var = "gene")
c5.c0.YvR$cluster <- 0


c5.c3.YvR <- FindMarkers(cluster5,
                         ident.1 = "Yap_3",
                         ident.2 = "RGFP_3",
                         verbose = FALSE,
                         assay = "SCT",
                         slot = "data",
                         min.pct = 0,
                         logfc.threshold = 0)
c5.c3.YvR <- rownames_to_column(c5.c3.YvR, var = "gene")
c5.c3.YvR$cluster <- 3


c5.c4.YvR <- FindMarkers(cluster5,
                         ident.1 = "Yap_4",
                         ident.2 = "RGFP_4",
                         verbose = FALSE,
                         assay = "SCT",
                         slot = "data",
                         min.pct = 0,
                         logfc.threshold = 0)
c5.c4.YvR <- rownames_to_column(c5.c4.YvR, var = "gene")
c5.c4.YvR$cluster <- 4

c5.c5.YvR <- FindMarkers(cluster5,
                         ident.1 = "Yap_5",
                         ident.2 = "RGFP_5",
                         verbose = FALSE,
                         assay = "SCT",
                         slot = "data",
                         min.pct = 0,
                         logfc.threshold = 0)
c5.c5.YvR <- rownames_to_column(c5.c5.YvR, var = "gene")
c5.c5.YvR$cluster <- 5
```
